# Experiments on Waste Heat Thermoelectric Generation for Passenger Vehicles

**DOI:** 10.3390/mi13010107

**Published:** 2022-01-10

**Authors:** Jianfei Chen, Wei Xie, Min Dai, Guorong Shen, Guoneng Li, Yuanjun Tang

**Affiliations:** 1 Zheneng Intelligent Energy and Technology Industrial Park, Huzhou 313100, China; cjf661121@163.com (J.C.); xiewei@zhenergy.com.cn (W.X.); dm13355828192@163.com (M.D.); staffshen2022@163.com (G.S.); 2 Department of Energy and Environment System Engineering, Zhejiang University of Science and Technology, Hangzhou 310023, China; 119037@zust.edu.cn

**Keywords:** waste heat recovery, thermoelectric generator, heat collection efficiency, overall efficiency, pressure drop

## Abstract

In order to utilize waste heat from passenger vehicles by a thermoelectric generator (TEG), a lab-scale TEG with a sufficient low-pressure drop was designed and tested. The waste heat from a 2.0 L petrol engine was simulated by using an air-circulation channel with an adjustable electric heater and a speed control motor. The TEG consisted of an integrated molding designed aluminum-finned heat collector, twenty thermoelectric modules, and a set of water-cooled heat sinks. Experiments were conducted in terms of power load feature, pressure drop, heat collection efficiency, thermoelectric efficiency and overall efficiency. It was found that the hot-end temperature was much lower (46.9%) than the flue gas temperature because the trade-off between fin area and pressure drop had to be considered. The obtained maximum electric power was 36.4 W, and the corresponding pressure drop was 36 Pa. The corresponding heat collection efficiency was 46.5%, and the thermoelectric efficiency was 2.88%, which agreed well with the theoretical prediction of 3.38%. As a result, an overall efficiency of 1.21% was reached. The present work firstly demonstrated a waste-heat-recovering TEG prototype with a balanced overall efficiency of over 1%, and a pressure drop of less than 50 Pa. On the other hand, the maximum electric power was difficult to fully extract. The charging power to a battery with a maximum power point tracking direct current–direct current converter was experimentally verified to work at a much higher conversion efficiency (15.3% higher) than regular converters.

## 1. Introduction

Nowadays, the recovery of waste heat for industrial conversion into electricity is among the major challenges facing humanity. A thermoelectric generator (TEG) is one of the promising candidates. The working principle of a TEG is the Seebeck effect, which directly converts temperature difference into electricity in a solid state. Owing to great advances of TE materials in recent years, TEGs with a high efficiency are expected to emerge in the near future. For example, SnSe has a figure-of-merit (*ZT*) value of 2.6 in high-temperature zones [1], and further improvements found that the *ZT* value could reach 1.34 in low-temperature zones [2]. Therefore, TEG has been studied in many applications. These applications include electricity generation in space exploration and in remote areas [3], waste heat recovery in automobiles, aircrafts, ships and industrial flue gas pipelines [4] and micro TEGs [5]. A brief literature review, focusing on recent TEG studies utilizing waste heat from passenger vehicles is provided, as follows.

Remeli et al. [4] proposed a concept of combined heat recovery and power generation using a heat-pipe-assisted TEG. Although the measured TE efficiency of 0.7% was relatively low, a heat exchanger effectiveness of 41% was reached. Kim, Negash and Cho [6] designed a direct contact TEG in order to remove the contact resistance between the TE modules and the heat resource. The electric power per TE module was 1.08 W. Li et al. [7] filled foam metals in the flue gas channel, aiming to improve the TEG performance, and the electric power per TE module was 2.75 W. However, the pressure drop was found to be considerable (10,000 Pa). Kim, Kwak and Kim [8] developed a hexagonal-shaped TEG for passenger vehicle applications, where a total of 18 TE modules were installed. The electric power per TE module was 5.44 W. These results were obtained when the hot-end temperature was 336 °C. The heat collection efficiency was measured to vary between 18% and 32.9%, and the pressure drop was between 400 Pa and 2000 Pa. A dynamic model of automobile TEG was developed by Lan et al. [9]. Numerical results revealed that a 20% average power output increase can be expected by optimizing the thermal contact conductance and the heat transfer coefficient of the heat collector. Massaguer et al. [10] performed detailed numerical investigations on an automobile TEG. They found that the maximum fuel economy value was only 0.18% with their TEG. The TEG performance utilizing flue gases and humidified flue gases were analyzed and compared using energy and exergy analysis by Zhao et al. [11]. Results found that the largest exergy destruction had different patterns for these two scenarios. Eddine et al. [12] found that the optimum clamping pressure for the TEG device was 0.35 MPa in their test-rig, which simulated the heat recovery of a marine engine. The measured TE efficiency varied between 0.26% and 0.4%. Zhao et al. [13] proposed a media-based TEG, and numerical results found that there was an optimal module area to maximize the output power when the exhaust heat exchange area was fixed. Marvão, Coelho and Rodrigues [14] proposed a gradient-based search method to optimize the fin structure inside a TEG for waste heat recovery in heavy-duty vehicles. Numerical results found that the thickness of the fins, wall ducts, electrical conductors and ceramic strips should be as small as possible. Recently, Tang et al. [15] developed a new type of high temperature heat pipe to assist TEG application, and experimental results found that the developed heat pipe was a promising method to improve the overall efficiency of the TEG. Brito et al. [16] applied phase change material to stabilize the hot-end temperature of the TEG, and solve the by-pass problem during high thermal-load events.

As revealed from the above analysis, recent studies have considerably augmented heat collection efficiency by installing foam metals into the gas channels [7], or increasing the gas velocity [8]. However, critical pressure drops, for example, 10 kPa [7] and 0.4~2 kPa [8], have been caused which, in return, lead to the performance degradation of the engine. Hence, further studies on automobile TEG are needed, particularly those focusing on the trade-off between the heat collection and pressure drop. In the present work, a new automobile TEG with a sufficient low-pressure drop, and attractive heat collection efficiency, was designed and tested. The gas flow rate in the present work was designed in accordance with a 2.0 L engine. Experiments were conducted in terms of power load features, pressure drop, heat collection efficiency, TE efficiency and overall efficiency. The obtained results showed that a minor pressure drop can be achieved while maintaining relatively large TE efficiency and overall efficiency. The contribution of the present work is to provide new experiment data and analysis in TEG applications with sufficient low-pressure drop.

## 2. Materials and Methods

### 2.1. Experimental Setup

**Figure 1:**
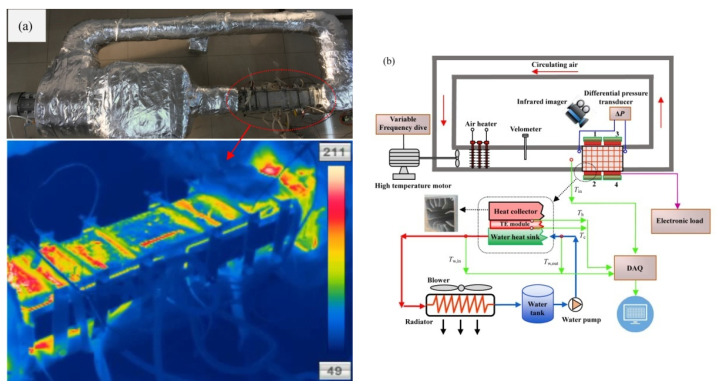
Experimental setup. (**a**) Photograph and thermal image of the TEG system. (**b**) Schematic of working principle and measuring system.

The schematic of the experimental setup is shown in Figure 1. The TEG system consisted of a square aluminum-finned heat collector, twenty TE modules, four water-cooled heat sinks, a water tank, a water pump and a heat radiator with blowers. The heat collector was mounted directly to the exit of the electric heater as part of the circulation channel. Dimensions of the heat collector were 450 mm [length] × 77 mm [inner width] × 77 mm [inner height], and 12 flat-plate fins with a thickness of 2.5 mm were designed. The distribution of the abovementioned fins adhered to the following procedures: (1) a cylinder-shaped hollow zone with a diameter of 38 mm was deliberately left, which acted as the bypass channel; (2) eight fins (four on each side) on two opposite sides of the inner wall of the heat collector were integrated, four fins were measured with a height of 16 mm, and the height of the other four fins was 23 mm; (3) four fins (two on each side) with a height of 19 mm on the other two opposite sides of the inner wall of the heat collector were arranged. A speed control motor was installed upstream of the electric heater, providing the forcing power of the air flow, and a variable frequency drive was used to adjust the motor speed. The heating power varied between 1 kW and 3 kW. The air velocity varied between 2.06 m/s and 8.11 m/s, which was measured with a Testo hot-wire velocimeter 405i with an accuracy of 5%. An infrared imager Dali T8 was used to capture the temperature distribution of the TEG system. A differential pressure transducer CHY-130 was installed to measure the pressure drop of the heat collector. The resolution of the thermal imager was 25 μm, and the accuracy of the differential pressure transducer was 0.5%.

A commercially available Bi_2_Te_3_-based TE module, type “TEP1-126T200”, with dimensions of 40 mm [length] × 40 mm [width] × 3.3 mm [thickness], was employed in the present TEG. According to the datasheet from the manufacturer, the internal electrical resistance of the TE module was 3.4 Ω, and the long-term working temperature was 200 °C. The TE efficiency was 4.9% under the conditions of hot-/cold-end temperatures of 200 °C and 30 °C, respectively. The height and cross-sectional area of the TE leg were 1.5 mm and 1.3 mm × 1.3 mm, and the thickness of the ceramic plate was 0.9 mm. Meanwhile, various physical properties were also provided from the manufacturers to predict the TE efficiency theoretically. Four heat sinks with dimensions of 200 mm [length] × 40 mm [width] × 11.8 mm [thickness] were used to cool down the cold-end of the TE module. A circular water channel with a diameter of 6 mm was designed inside the heat sink. The water channel was equidistantly distributed in an M shape, and the length of the four parallel sub-channels were 180 mm. Four identical groups of TE module were installed in two opposite sides (with dense fins) of the heat collector, which is shown in Figure 1b. Group-1 and Group-2 were placed upstream of Group-3 and Group-4, respectively. The only difference between Group-1 (Group-3) and Group-2 (Group-4) was the measuring holes for the differential pressure transducer. In other words, two pressure measuring holes were designed ahead of Group-1 and behind of Group-3, respectively. Five TE modules were installed in each group. Therefore, a total of twenty TE modules were installed. In the present work, the output voltage of TEG was designed for batteries with a rated voltage of 24 V. Hence, ten TE modules in each side of the heat collector were wired in series, and two sides were wired in parallel. Thus, the open circuit voltage of the TEG could be larger than 50 V, which helped to extract as much electric power as possible during battery charging.

Eleven type-K thermocouples were installed to measure the inlet air temperature, hot-end temperatures, cold-end temperatures, inlet water temperature and outlet water temperature, respectively. The accuracy of the thermocouples was 0.5%, and an Agilent-34970A data-acquisition (DAQ) instrument was used to record the temperature signals. The hot-/cold-end temperatures are measured with type-K patch thermocouples, which had a sufficient large contact area to compensate the thermal-conduction-induced measuring errors. Several “ears” were integrally molded with the heat collector and heat sink, providing installation locations for the abovementioned patch thermocouples. Furthermore, the abovementioned patch thermocouples were fastened with aluminum rivets. The errors caused by the thermal resistance of “ears” were taken into consideration when measuring hot-/cold-end temperatures. A Prodigit 3311F electronic load was used to explore the power load feature, and its accuracy was 0.5%. In order to compare the performance of different kinds of DC–DC converters (DDCs), two regular DDCs, type ZS-Q5, and type DKP6012, and one maximum power point tracking (MPPT) DDC, type YF-BKT80V15A, were used.

The atmosphere and inlet water temperatures during experiments were 22 °C and 19 °C, respectively. Three heating powers, i.e., 1 kW, 2 kW and 3 kW, and four flue gas velocities, i.e., 2.06 m/s, 4.73 m/s, 6.48 m/s and 8.11 m/s, were selected in the experiments. Therefore, 12 running conditions were chosen, and 15 load resistances were explored for each running condition. As a result, a total of 180 experimental cases were conducted. The working conditions for water pump and the heat radiator remained unchanged during experiments. The heat flux entering into the heat collector was obtained by an electric kilowatt-hour meter on the basis of neglecting the heat dissipation from the outer surface of the channel between the electrical heater and the heat collector. The neglect of heat dissipation was reasonable, because insulations were applied and the distance between the electrical heater and heat collector was limited. This was confirmed by the thermal image, as shown in Figure 1. No obvious high temperature zones were found with the infrared imager, except the testing TEG. The electric kilowatt-hour meter was provided by the state grid Co., Ltd., and the accuracy was 0.1%.

### 2.2. Data Reduction

Only part of the heat in the flue gases can be extracted by the heat collector. Therefore, the heat collection efficiency is defined as:

(1) $$\eta_{\mathrm{heat}}=\frac{Q_{\mathrm{HC}}}{P_{\mathrm{in}}}$$

where *P*_in_ is the heating power, which represents the heat flux of flue gases entering into the heat collector. *Q*_HC_ denotes the heat flow rate extracted by the heat collector. Furthermore, only partial *Q*_HC_ was responsible for power generation by TE modules, while others were dissipated by convection and radiation. The actual heat flux extracted by TE modules, *Q*_TE_, can be obtained as follows:

(2) $$Q_{\mathrm{TE}}=Q_{\mathrm{HC}}-Q_{\mathrm{conv}}-Q_{\mathrm{rad}}$$

where *Q*_conv_, and *Q*_rad_ are the heat loss rates by convection and thermal radiation, respectively. Therefore, the effective heat collection efficiency can be defined as follows:

(3) $$\eta_{\mathrm{heat},\mathrm{eff}}=\frac{Q_{\mathrm{TE}}}{P_{\mathrm{in}}}$$

TE modules converse directly part of *Q*_TE_ to electricity. Therefore, TE efficiency and overall efficiency can be defined as follows:

(4) $$\eta_{\mathrm{TE}}=\frac{P_{\max}}{Q_{\mathrm{TE}}}$$

(5) $$\eta_{\mathrm{sys}}=\frac{P_{\max}}{P_{\mathrm{in}}}$$

where *P*_max_ is the obtained maximum electric power. Hence, the TE efficiency must be larger than the overall efficiency, and satisfy the following equation.

(6) $$\eta_{\mathrm{sys}}=\eta_{\mathrm{heat},\mathrm{eff}}\eta_{\mathrm{TE}}$$

The TE efficiency can be predicted theoretically with the following equation [17]:

(7) $$\eta_{\mathrm{TE}}=\frac{T_{h}-T_{c}}{T_{h}}{\left\{{\left(1+2rw\right)}^{2}\left[2-0.5\left(\frac{T_{h}-T_{c}}{T_{h}}\right)+\left(\frac{4}{ZT_{h}}\right)\left(\frac{1+n/L}{1+2rw}\right)\right]\right\}}^{-1}$$

where *T*_h_ is the hot-end temperature, and *T*_c_ denotes the cold-end temperature. The TE efficiency is mainly controlled by the figure-of-merit (*Z*), which is defined as follows [18]:

(8) $$Z=\frac{\alpha^{2}}{k\rho }$$

where *α, ρ* and *k* are the Seebeck coefficient, electrical resistivity and thermal conductivity, respectively. *r* and *w* are the thermal contact ratio and the dimensional ratio of TE module, respectively. *n* denotes the electrical resistivity ratio. For the present study, *w* = 0.516. *r* = 0.2, *n* = 0.1 [18]. *L* is the TE leg length, and *L* = 1.5 mm. The *ZT*_ave_ value in the present experimental cases varied between 0.80 and 0.85.

## 3. Results and Discussions

### 3.1. Temperature

**Figure 2:**
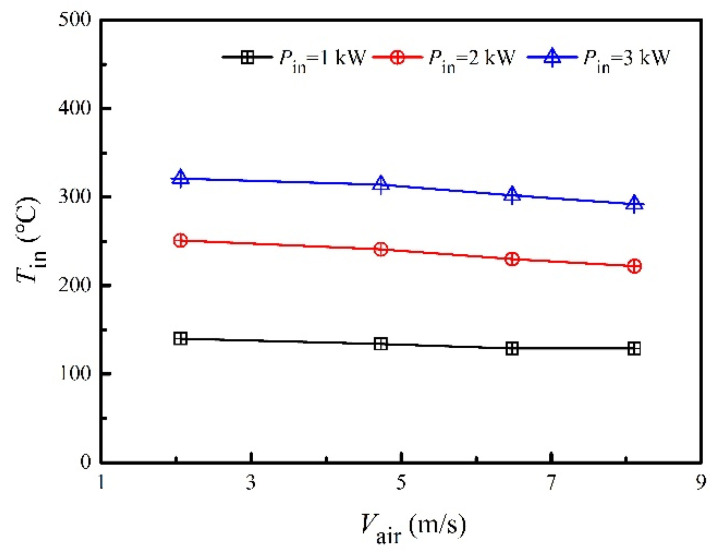
Inlet air temperatures of the heat collector under various heating powers and air velocities.

Figure 2 presents the inlet air temperature of the heat collector under various heating powers and air velocities. The air temperature was considerably high, even at the heating power of 1 kW, which was benefited by the design of the air-circulation channel. The slightly decreasing trend of inlet air temperature as the air velocity increased was caused by the enhancement of convective heat transfer between the flowing air and channel walls. The average inlet air temperatures were 133 °C, 236 °C and 307 °C for the heating power of 1 kW, 2 kW and 3 kW, respectively, and the corresponding differences due to the air velocity were 11 °C, 28 °C and 29 °C, respectively. The flue gas temperature at the exit of the engine can be higher than 300 °C [6]. However, the installation of a three-way-catalytic-converter and a muffler between the TEG and the engine can considerably decrease the flue gas temperature. Moreover, the contamination of fins in real applications will increase the heat transfer thermal resistance between the flue gas and heat collector. Thus, developing TEGs that work in clean flue gases with a flue gas temperature of approximately 300 °C is appropriate. The distance between the electric heater and the heat collector was short, compared with the whole length of the channel. Therefore, the input power to the heat collector was assumed to be the electric heating power. In other words, the input power to the heat collector was assumed to be 1 kW, 2 kW and 3 kW, respectively.

**Figure 3:**
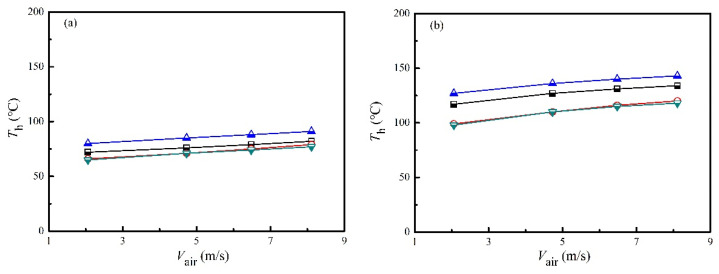

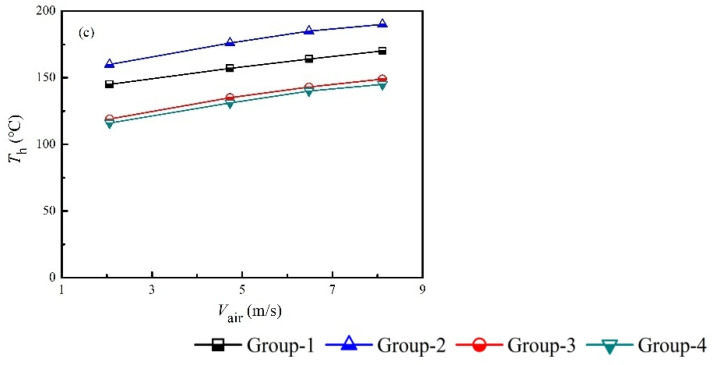
Hot-end temperatures of different groups under various heating powers and air velocities. (**a**) *P*_in_ = 1 kW. (**b**) *P*_in_ = 2 kW. (**c**) *P*_in_ = 3 kW.

**Figure 4:**
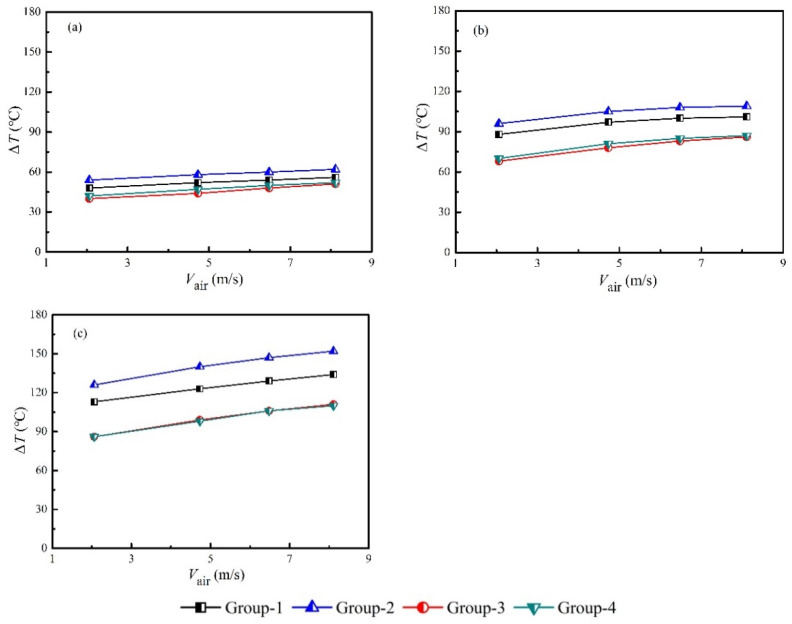
Temperature differences of different groups under various heating powers and air velocities. (**a**) *P*_in_ = 1 kW. (**b**) *P*_in_ = 2 kW. (**c**) *P*_in_ = 3 kW.

The hot-end temperatures and corresponding temperature differences of different groups of TE modules under various heating powers and air velocities were shown in Figure 3 and Figure 4. As shown in Figure 3, the hot-end temperature increased slightly with the air velocity, which was also caused by the enhanced convective heat transfer between the flowing air and the heat collector. It was interesting to found that the hot-end temperatures were quite different for Group-1 and Group-2, whereas the hot-end temperatures for Group-3 and Group-4 were close to each other. A possible reason for this phenomenon was the uneven flow field, due to the elbow upstream of the heat collector, which was shown in Figure 1. An important finding was the difference between the hot-end temperature and the inlet air temperature, i.e., the hot-end temperatures were considerably lower than the inlet air temperature. The average hot-end temperatures were 76.9 °C, 121.3 °C and 151.6 °C for the heating power of 1 kW, 2 kW and 3 kW, respectively. This implied that an average temperature decreases of 108.7 °C was found from flue gas temperatures to hot-end temperatures. Advanced methods, such as thermosyphon cooling [19], could be helpful to narrow the abovementioned gap. As a result, an essential problem of applying TEG to recover waste heat from flue gases should be the heat collection technology. A possible method to increase the hot-end temperature under a specific flue gas temperature is to increase the fin density. However, this is subject to the pressure drop problem, which will be discussed in Section 3.5. Balancing the heat collection efficiency and pressure drop is one of the major tasks in developing TEGs to utilize waste heat from engines.

The temperature differences increased with the heating power and air velocity, which was shown in Figure 4. The average temperature differences were 51.1 °C, 90.1 °C and 116.6 °C when the heating power was set at 1 kW, 2 kW and 3 kW, respectively. The corresponding differences due to the air velocities and different groups were 12.8 °C, 25.8 °C and 42.25 °C, respectively. Therefore, obvious differences of electric power output were expected, which will be discussed in Section 3.2.

### 3.2. Power Load Feature

**Figure 5:**
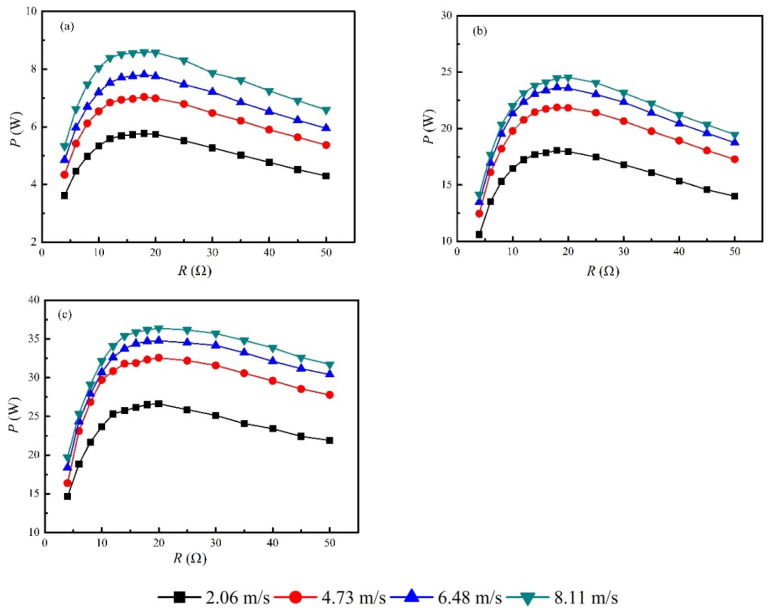
Power load feature of total electric power under various heating powers and air velocities. (**a**) *P*_in_ = 1 kW. (**b**) *P*_in_ = 2 kW. (**c**) *P*_in_ = 3 kW.

The electric powers generated by the TEG system at different load resistances under various heating powers and air velocities were shown in Figure 5. Air velocity was important in the augmentation of electric power output under a fixed heating power. For example, the maximum electric power at 8.11 m/s was 36.4 W at *P*_in_ = 3 kW. However, the maximum electric power was only 26.6 W at 2.06 m/s, under the same heating power, which means that a 26.9% of downgrade was reached. On the other hand, power load tests showed that the optimized load resistance was located at 20 Ω, indicating that the internal resistance of each TE module was 4 Ω. This was slightly larger than the data (3.6 Ω) provided by the manufacture, which was caused by the increased temperature compared with the room temperature. The maximum electric powers were 8.6 W, 24.5 W and 36.4 W when the heating power was set at 1 kW, 2 kW and 3 kW, respectively. This implies that the inlet air temperature was another important parameter for the power generation of the TEG system. In order to increase the electric power output, both the temperature and the velocity of the flue gases should be as large as possible. However, these two parameters cannot be optimized in waste heat recovery applications because they are boundary conditions. The power generation densities were 268.7 W/m^2^, 765.6 W/m^2^ and 1137.5 W/m^2^ based on the area of the TE modules when the heating power was set at 1 kW, 2 kW and 3 kW, respectively. It seems that these power generation densities were obviously larger than that (~150 W/m^2^) of commercial solar plates. However, using the internal area of the heat collector (0.151 m^2^) to calculate the power generation density led to new values of 57 W/m^2^, 162 W/m^2^, and 241 W/m^2^, respectively. As a consequence, these power generation densities turned to be comparable to that of solar plates. Nevertheless, TEG could be the most promising technology to convert waste heat into electricity in distributed small-scale engines, such as engines in passenger vehicles and inland vessels. The major reasons include its compactness and solid-state energy-conversion process, which are essentially required, as space is limited in the abovementioned energy systems.

### 3.3. Efficiency

**Figure 6:**
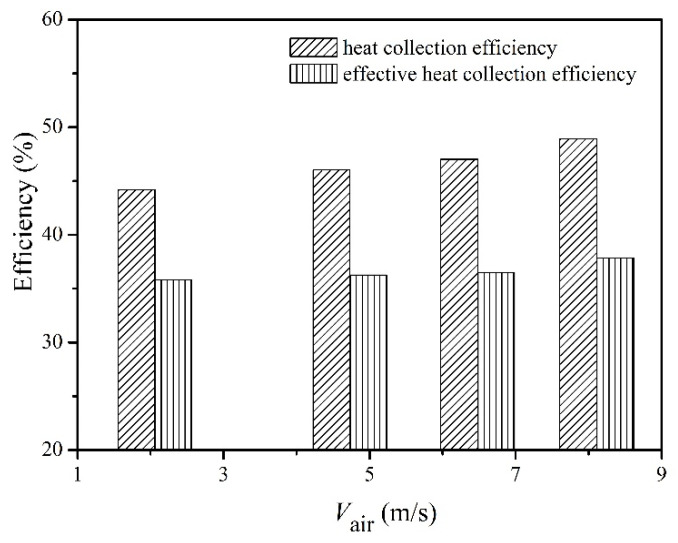
Heat collection efficiency when the heating power was 3 kW.

As mentioned above, one of the essential issues in applying TEG technology in utilizing waste heat is the performance of the heat collector. Therefore, it is important to discuss the heat collection efficiency of the present TEG, which is shown in Figure 6. In the present work, no insulation was applied to the heat collector. As a result, a certain amount of heat flux extracted from the heated air by the heat collector was dissipated to the surroundings through natural convections and thermal radiations. Therefore, only part of the heat passed through the TE modules.

As shown in Figure 6, the average heat collection efficiency according to Equation (1) was 46.5%, and the average effective heat collection efficiency decreased to 36.6%, according to Equation (3). This was consistent with previous works, i.e., 32.9% in Kim’s work [8]. Increasing fin density could help to improve the heat collection efficiency, but it is subject to the pressure loss problem. Increasing the length of the heat collector could be another way to augment the heat collection efficiency. However, as shown in Figure 4, the temperature differences for Group-3 and Group-4 were obviously lower than those of Group-1 and Group-2. Therefore, the temperature difference will become even smaller as the length of the heat collector increases. As a consequence, minor improvements can be obtained when increasing the length of the heat collector.

**Figure 7:**
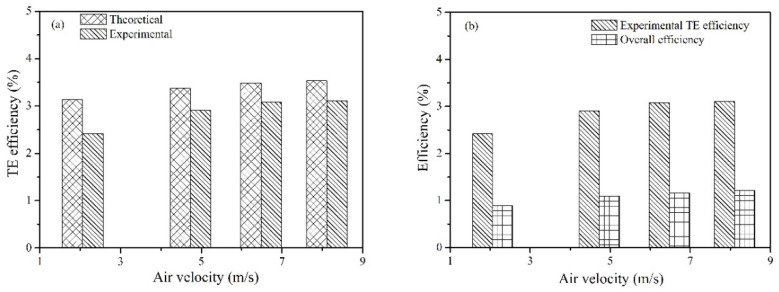
Conversion efficiencies when the heating power was 3 kW; (**a**) comparison between theoretical TE efficiencies and experimental results; (**b**) comparison of TE efficiencies and overall efficiencies.

The measured TE efficiency according to Equation (4) can be used to reveal the effectiveness of TE modules, which should be close to the theoretically predicted result according to Equations (7) and (8). The experimental TE efficiency, the predicted TE efficiency and the overall efficiency are shown in Figure 7. The average measured TE efficiency was 2.88%, whereas the predicted TE efficiency was 3.38%, which was based on the temperature-dependent properties of the TE materials. Many parameters, including hot/cold-end temperatures, electric power, the convective heat transfer coefficient, thermal contact ratio, and the electrical resistivity ratio and wiring method of the TEG, affect the consistency between the measured TE efficiency and theoretical prediction. The wiring method could be a major reason for the abovementioned difference in TE efficiency. As shown in Figure 4, the temperature differences were different in different groups of TE modules, indicating that a certain amount of electric power was lost when wiring the TE modules in parallel, which was demonstrated in previous works [20]. The maximum overall efficiency was 1.21% at the air velocity of 8.11 m/s when the heating power was set at 3 kW. Based on above analysis, two important parameters were revealed to be essential when applying TEG technology to recover waste heat, i.e., heat collection efficiency and TE efficiency. As mentioned in Equation (8), the TE efficiency was determined by the physical properties of the TE materials, which relies on the innovation of new TE materials [1]. Otherwise, multi-stage TE modules [21] and combined photovoltaic-thermoelectric generation [22] were possible methods to increase the overall efficiency. Note that the abovementioned overall efficiency does not take the electric power consumption of the water pump and blower into consideration, due to the fact that the cooling systems differed from each other in real applications. In the present work, a total electric power of 10.6 W was consumed by the water pump and blowers.

### 3.4. Influence of MPPT Technology on Electricity Output

**Figure 8:**
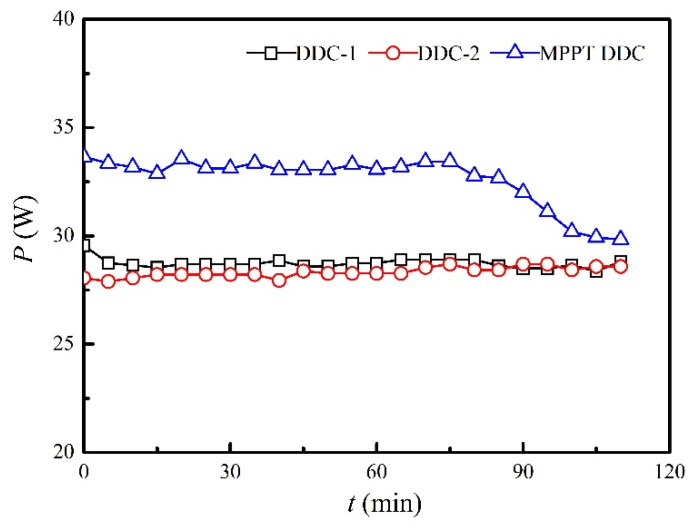
Comparison of charging power between regular DDCs and the MPPT DDC when the heating power and air velocity were set at 3 kW and at 8.11 m/s, respectively.

In real applications, a battery has to be employed to balance the power generation and electric output. Therefore, a DDC has to be used. Though MPPT DDCs are suggested in many references [23], a direct comparison between MPPT DDC and regular DDC is still lacking, specially presenting their charging curves into batteries. Figure 8 shows the experimental measured performances of two regular DDCs and a MPPT DDC. The internal resistance of the battery varies with time; therefore, the maximum electric power (36.4 W) cannot be reached. As shown in Figure 8, the MPPT DDC performs better than regular DDCs. The average charging power into the battery in the first 60 minutes was 33.2 W for the MPPT DDC, whereas the average charging powers were 28.8 W and 28.2 W for DDC-1 and DDC-2, respectively. This implied that an improvement of 15.3% was achieved, which was consistent with a previous work, i.e., 14.5% of improvement reported in Yu and Chau’s work [24]. After 80 min of charging, the battery accepted less electric power, and the charging power dropped to a lower value for MPPT DDC, whereas the charging powers for regular DDCs maintained unchanged, because the total amount of charged electric power was less than that with MPPT DDC. In other words, the shorter time needed to reach a saturated charging mode denoted a higher conversion efficiency of the DDC. The conversion efficiency for MPPT DDC was 91.2%, whereas the average conversion efficiencies were 79.2% and 77.5% for DDC-1 and DDC-2, respectively, which was consistent with our previous works [25]. Therefore, MPPT DDC is recommended in future studies.

### 3.5. Pressure Drop

**Figure 9:**
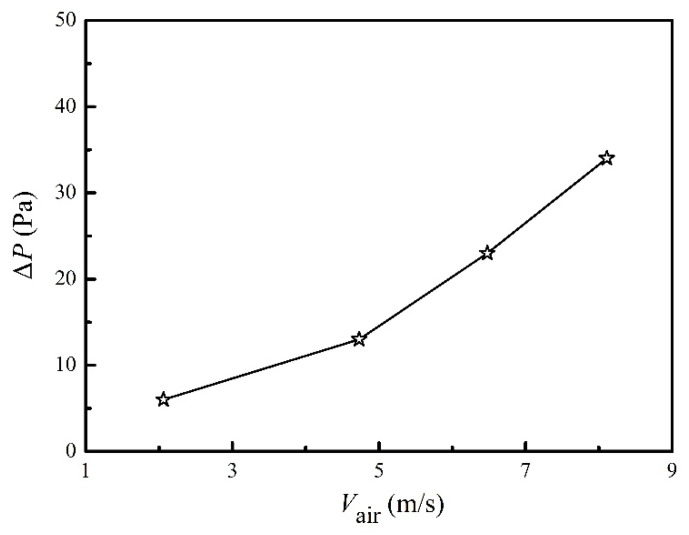
Pressure drops at different air velocities when the heating power was 3 kW.

In field applications, pressure drop has to be considered combining with the heat collector performance. In the case of adding a heat collector downstream of the exit of the waste heat resource, too large a pressure drop will cause failure in the upstream equipment. The pressure drop is directly linked to the gas velocity and the fin design. The measured pressure drop of the present heat collector is shown in Figure 9. Heating power had a minor influence on the pressure drop, which is not shown in Figure 9. The maximum pressure drop was 36 Pa, and it decreased further as the air velocity became smaller. This pressure drop was much smaller than previous studies, i.e., 10,000 Pa in Li’s work [7], 400–2000 Pa in Kim’s work [8], and 380 Pa in Kim’s work [6]. Table 1 presents a comparison between the results in this work and previous reports. The moderate heat collection efficiency of 46.5% and the notably low-pressure drop of 36 Pa were achieved through proper fin design and the integrity of the heat collector. The trade-off between the heat collection and the pressure drop could be achieved by considering the following aspects. (1) Dense fins on targeted surfaces. In the present work, four fins were designed in the surfaces where TE modules were installed, whereas the other two surfaces only had two fins. (2) A heat collector with an integrated molding design. The integrity of the aluminum tube in this work could transfer heat to the TE modules from every part of the heat collector, because the thermal conductivity (~180 W/m^2^-K) was much higher than that of TE modules (~1.5 W/m^2^-K). (3) A built-in by-pass channel inside the heat collector. The central part of the heat collector in the present work, with a diameter of 40 mm, was kept empty without any fins, helping to decrease the pressure drop. In future works, certain disturbers could be placed alternately in this empty space to disturb the thermal boundary layers of the fins, so as to increase the heat collection efficiency. Besides, designing pin-fins inside the heat collector and separating flue gases to multiple streams are possible directions to improve heat collector performance.

## 4. Conclusions

A lab-scale thermoelectric generator (TEG) utilizing waste heat from flue gases of passenger vehicles was designed and tested to study the heat collection efficiency, thermoelectric efficiency, overall efficiency, and electric power extracting efficiency under the condition of a sufficient low-pressure drop. The following conclusions can be drawn based on above analysis:

(1) The integrated molding design of heat collector with appropriate fin distribution was proven to be able to achieve a considerably low-pressure drop, while keeping a relatively high heat-collection efficiency. In the present TEG prototype, the pressure drop was 36 Pa, which is much lower than those of previous studies. Meanwhile, the heat collection efficiency of 46.5% was comparable with previous works;
(2) The overall efficiency of the TEG system was relatively low (1.21% in maximum), which was determined by the heat collection efficiency and the thermoelectric efficiency. The experimental thermoelectric efficiency was 2.88%, which was close to the predicted value of 3.38%. Essential issues when applying TEG to recover waste heat include optimizing the heat collector and TE materials;
(3) Maximum power point tracking (MPPT) direct current–direct current converters (DDC) should be used because they performed much better than regular converters. In the present work, the conversion efficiency of the MPPT DDC was 91.2%, whereas the average conversion efficiency was lower than 80% for regular DDCs.
(4) The insufficient heat transfer nature between flue gases and the solid-heat collector must be improved in the future work. As revealed in the present work, the average hot-end temperatures were 46.9% lower than the inlet flue gas temperatures. This reduced the application potential of TEG for utilizing waste heat from passenger vehicles. Thermosyphon, staggered pin-fins and appropriate flow perturbations are possible research directions.

## Figures and Tables

**Table 1:** Performance comparisons with various previous studies.

Reference	Overall Efficiency (%)	Heat Collection Efficiency (%)	Pressure Drop (Pa)
Kim, Negash and Cho (2017) [6]	1.4–2.0	− *	380
Li et al. (2017) [7]	0.8~1.2	−	2000~10,000
Kim, Kwak and Kim (2018) [8]	1.3~2.6	32.9	400~2000
Comamala et al. (2019) [26]	1.08	−	5400
present	1.21	46.5	36

* Denotes unknown or not reported.

## Data Availability

The data presented in this study are available on request from the corresponding author.

## Nomenclature

*k*	thermal conductivity (W/m-K)
*L*	length of the TE leg (m)
*n*	electrical resistivity ratio (m)
*P*	load power (W)
*Q*_conv_	heat loss rate by convections (W)
*Q*_HC_	heat flow rate extracted by heat collector (W)
*P*_in_	heating power (W)
*P*_max_	maximum electric power generated by the TEG system (W)
*Q*_rad_	heat loss rate by thermal radiations (W)
*Q*_TE_	heat flow rate passing through the TE modules (W)
Δ*P*	pressure drop (Pa)
*r*	thermal contact ratio (dimensionless)
*R*	load resistance (Ω)
*T*_ave_	average temperature of hot and cold ends (°C), *T*_ave_ = (*T*_h_ + *T*_c_)/2
*t*	time (minute)
*T*_c_	cold-end temperature (°C)
*T*_h_	hot-end temperature (°C)
*T*_in_	inlet air temperature (°C)
*T*_w,in_	inlet cooling water temperature (°C)
*T*_w,out_	outlet cooling water temperature (°C)
Δ*T*	temperature difference (°C), Δ*T* = *T*_h_ − *T*c
*V*_air_	air velocity (m/s)
*w*	ratio of ceramic thickness to the length of TE leg (dimensionless)
*Z*	figure-of-merit (1/K)
*α*	Seebeck coefficient (V/K)
*ρ*	electrical resistivity (Ωm)
*η*_heat_	heat collection efficiency (%)
*η*_heat,eff_	effective heat collection efficiency (%)
*η*_sys_	overall efficiency (%)
*η*_TE_	TE efficiency (%)
DAQ	data acquisition
DDC	DC-DC converter
MPPT	maximum power point tracking
TE	thermoelectric
TEG	TE generator
